# A Sulfur Hexafluoride Sensor Using Quantum Cascade and CO_2_ Laser-Based Photoacoustic Spectroscopy

**DOI:** 10.3390/s101009359

**Published:** 2010-10-18

**Authors:** Mila Rocha, Marcelo Sthel, Guilherme Lima, Marcelo da Silva, Delson Schramm, András Miklós, Helion Vargas

**Affiliations:** 1 Laboratório de Ciências Físicas LCFIS, Universidade estadual do Norte Fluminense UENF,Av. Alberto Lamego 2000, Campos dos Goytacazes, Rio de Janeiro, Brazil; E-Mails: mila_vieira16@hotmail.com (M.R.); guiuenf@yahoo.com.br (G.L.); mgs@uenf.br (M.S.); delson@uenf.br (D.S.); vargas@uenf.br (H.V.); 2 Fraunhofer Institute for Building Physics, Stuttgart, Germany; E-Mail: andreas.miklos@urz.uni-heidelberg.de

**Keywords:** sulfur hexafluoride, photoacoustic spectroscopy, sensors

## Abstract

The increase in greenhouse gas emissions is a serious environmental problem and has stimulated the scientific community to pay attention to the need for detection and monitoring of gases released into the atmosphere. In this regard, the development of sensitive and selective gas sensors has been the subject of several research programs. An important greenhouse gas is sulphur hexafluoride, an almost non-reactive gas widely employed in industrial processes worldwide. Indeed it is estimated that it has a radiative forcing of 0.52 W/m^2^. This work compares two photoacoustic spectrometers, one coupled to a CO_2_ laser and another one coupled to a Quantum Cascade (QC) laser, for the detection of SF_6_. The laser photoacoustic spectrometers described in this work have been developed for gas detection at small concentrations. Detection limits of 20 ppbv for CO_2_ laser and 50 ppbv for quantum cascade laser were obtained.

## 1. Introduction

Air pollution is one of the main environmental problems of the XXIst Century. After the publication of the Intergovernmental Panel on Climate Changes (IPCC) Report in 2007 [1], global warming is considered to be the responsible for climate changes in the planet [2–10] and for bringing significant consequences to human society.

The same Report highlights sulfur hexafluoride (SF_6_) as an important greenhouse gas, as it has a radiative forcing of 0.52 W/m^2^, an atmospheric lifetime of 3,200 years and a Global Warming Potential (GWP) of 23,900. Sulfur hexafluoride is almost non-reactive and therefore it is widely employed for insulation and current interruption purposes in electric power transmission and distribution equipment. It is also used in the magnesium industry to protect molten magnesium from oxidation and potential violent burning, in semiconductor manufacturing to create circuitry patterns on silicon wafers and as a tracer gas for leak detection [11].

In trace-gas analysis of chemical species in the atmosphere, besides sensitivity and selectivity the response time is of increasing interest for the real-time detection of temporal concentration changes. In order to achieve the necessary sensitivity and selectivity, the use of high-resolution laser techniques in IR and near-IR (NIR) fingerprint region is of special interest. The methodologies based on the photothermal techniques, mainly photoacoustic spectroscopy, have suitable characteristics for trace gas detection. Actually several laser-based methods have been reported because they are very sensitive [12–14]. For instance, photoacoustic spectroscopy is widely used in the detection of several gases in the ppbv and sub-ppbv concentration range [15–19].

A homemade CO_2_ laser photoacoustic spectrometer has been developed to monitor gas emissions of several sources [20]. A continuous wave CO_2_ infrared laser tunable over 80 different lines, between 9.2 and 10.6 μm, has been employed at the emission line of 10P(16) as excitation source for sulfur hexafluoride gas detection [21–23].

With the recent development of quantum-cascade lasers (QCLs), compact solid-state radiation sources are available, covering the important infrared (IR) region with specific molecular absorption lines. In addition, spectral regions known as atmospheric windows can be selected in which water vapor has a very low absorption coefficient. Another important advantage of QCLs in practical applications is that they work at near room temperature, whereas diode lasers such as lead salt lasers, which emit in the fundamental IR region, have to be cryogenically cooled.

Recent applications of QCLs clearly indicate their potential as tunable light sources in the mid-infrared, especially between 3 and 13 μm, with strong fundamental absorption bands. Current interest is based on the lack of other convenient coherent laser sources. In fact, it can be expected that QCLs will open new possibilities for real-time diagnostics of various molecular species in the 3–5 μm and 8–13 μm atmospheric windows [24].

Pulsed quantum-cascade distributed-feedback (QC-DFB) lasers provide quasi room temperature operation, combined with high spectral selectivity and sensitivity, real-time measurement capabilities, robustness, and compactness. For this reason, QCLs are ideal for the development of compact trace gas analyzers that are also suitable for field measurements. In recent years the detection of a series of important trace gases has been demonstrated with these devices [24–29].

In this work, we report on PA measurements of sulfur hexafluoride with two homemade Laser Photoacoustic Spectrometers, one equipped with a QC laser and another one with a CO_2_ laser, aiming to make comparisons of performance between traditional CO_2_ lasers (gas lasers) and new QC lasers (semiconductor lasers) for the detection of sulphur hexafluoride. In order to accomplish it, detection limit measurements were carried out. A value of 20 ppbv was achieved for the CO_2_ laser system and a value of 50 ppbv was achieved for the QC laser system. The motivation of our research comes from the necessity for simple, sensitive, and spectrally selective devices capable of measuring this important greenhouse gas at trace levels.

## 2. Methodology

### 2.1. CO_2_ Laser Photoacoustic Spectroscopy

**Figure 1.**
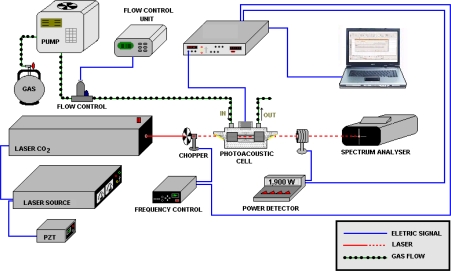
Scheme of the photoacoustic experimental setup.

**Figure 2.**
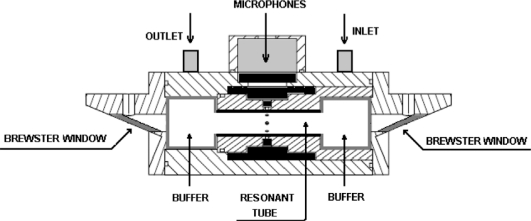
Diagram showing the design of the resonant photoacoustic cell used.

The gas samples were analyzed with a technique based on the photoacoustic method (Figure 1). In conventional absorption spectroscopy, one measures the absorption of the radiation power transmitted through the sample. On the contrary, in photoacoustic spectroscopy, the absorbed power is determined directly via its heat and hence the sound produced in the sample. Actually, several laser-based methods have been reported because they are very sensitive, as for instance photoacoustic and cavity-ring-down spectroscopy [30,31]. Photoacoustic spectroscopic methods offer important advantages with respect to contaminant gas monitoring. This technique is based on pressure changes in the gas sample, which is induced by ro-vibrational excitation of molecules and, subsequent; relaxation by collisions (heat). The pressure change is detected by one or more microphones placed inside a resonator pipe of a resonant photoacoustic cell (Figure 2), through which, the air sample; containing the molecules under consideration was flown. An acoustic signal is produced at the resonance frequency of about 2,400 Hz of our resonant cell, by a chopper modulation of the excitation laser beam. This resonance frequency value corresponds to the first longitudinal vibration mode. Our photoacoustic resonator is 67 mm long and has 18 mm in diameter.

The measurement was performed initially using a 1.1 ppmv certified gas mixture of ethylene in N_2_ flowing into the cell at a rate of 5 L/h. The ethylene gas is used as a calibrator in CO_2_ laser photoacoustic spectroscopy. The acoustic signal is detected by a microphone that generates an electric signal. This electric signal, in turn, is pre-amplified and then detected by a Lock-In amplifier (Stanford SR850). The Lock-In response is registered in a microcomputer. A continuous wave CO_2_ infrared laser (Lasertech Group Inc.,—LTG, model LTG150 626G), tunable over about 80 different lines between 9.2 and 10.6 μm and delivering a power of 1.9 W at the emission line of 10P(14), was employed as the excitation source. At this power level, no saturation effect of the photoacoustic signal was observed. The CO_2_ laser lines can be swept by a step motor controlled by the microcomputer. Within this spectral region many small molecules show a unique fingerprint. The photoacoustic instrument used in this work has been developed for gas detection at small concentrations. All measurements were made at room temperature.

Calibration and sensitivity measurements of our photoacoustic cell were performed obtaining the cell constant C in the Equation (1) below. This was carried out taking a certified mixture of 1.1 ppmv of ethylene in N_2_ and diluting it in nitrogen until the lowest detected concentration of 16 ppbv. Thus, a calibration curve relating the PA signal and the ethylene concentration was obtained. As this function has a linear behavior we can extend this linearity to ppmv levels. This calibration line follows the expression:

(1) $$S_{i}=C.P_{i}N_{\mathrm{tot}}c_{\mathrm{ref}}\sigma_{i}$$

The constant C is the so-called cell constant and it depends, like the detection sensitivity, on the cell geometry, the microphone responsivity and on the nature of the acoustic mode [32,33]. The absorption cross section σ of ethylene is well known at the 10P (14) (k = 949.51 cm^−1^) CO_2_ laser line (σ = 170 × 10^−20^ cm^2^/molecules). *P_i_* represents the laser power at wavelength *λ_i_* and *c_ref_* is the concentration of the gas. *N_tot_* is the total number density of molecules in the mixture and was considered to be typically 2.5 × 10^19^molecules/cm^−3^ at room temperature [32]. Hence, the C constant value was then obtained from the Equation (1), which yielded C = 40.2 V cm/W.

### 2.2. QC Laser Photoacoustic Spectroscopy

**Figure 3.**
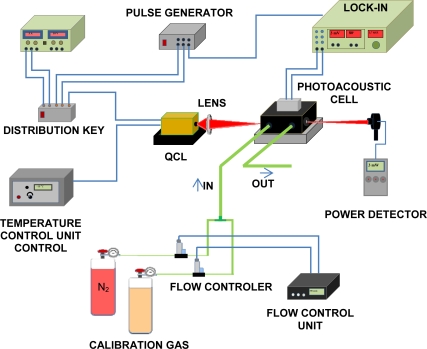
Experimental set up.

The experimental set up employed for the detection limit determination of the analyzed gas is illustrated in Figure 3. As radiation source, a pulsed quantum cascade laser furnished by Alpes Lasers was used. The laser (model #sb186 DN) emits in the range of 10.51–10.56 μm and can reach a power of 3.7 mW.

The laser wavelength can be turned over the given range by diode temperature, which is determined by a temperature control unit. The pulsed QCL light beam, with a repetition rate of 400 kHz and a pulse duration of 50 ns (duty cycle of 2%), was gated by an external transistor-to-transistor logic (TTL) signal at 3.8 kHz to excite the first longitudinal acoustic mode of the resonant differential photoacoustic cell [18,19]. A germanium lens (focus ∼30.7 mm and diameter ∼10.35 mm) was employed to focus the QCL radiation through the cell. The differential cell has two resonant cylindrical tubes (5.5 mm in diameter and 4 cm in length) on whose edges are arranged acoustic buffers which reduce noise caused by gas turbulence and background signal produced by the heating in the cell windows when these are exposed to the radiation. The gas flow streams through both pipes and noise and background were equally detected by the microphones placed on each of them, but only the microphones placed in the tube crossed by the laser could detect the pressure changes induced by the absorption of modulated radiation. Thus the photoacoustic signal is obtained as the difference of the microphones signals measured in the two tubes.

The laser power was monitored by a power detector (OPHIR, 3A-SH-ROHS) and the gas flows were controlled by two electronic mass flow controllers (model MKS 247, 50 sccm and 300 sccm). The PA data analysis was performed by the lock-in technique using a lock-in amplifier (model Stanford SR 850 DSP) with a time constant of 300 ms. The theoretical model adopted was the same as used in the CO_2_ laser photoacoustic system.

## 3. Results and Discussion

**Figure 4.**
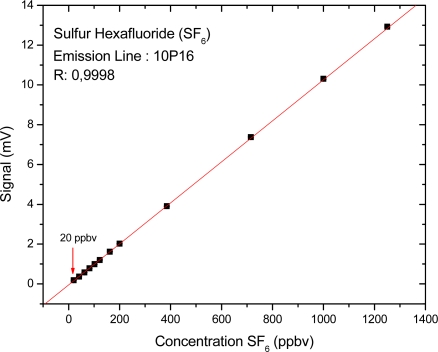
Calibration curve for sulfur hexafluoride.

The continuous wave infrared CO_2_ laser, delivering a power level of 1.5 W, was employed as an excitation source at the emission line of 10P (16), λ = 10.5 μm. The initial concentration of 5 ppmv of SF_6_ was diluted with pure nitrogen (zero gas) down to the lowest concentrations detected by the system. The acoustic and electronic noises were determined by blocking the laser light while keeping all other devices running. The value of the noise signal was typically 30 μV. As expected, a linear dependence of the photoacoustic signal on the sulfur hexafluoride concentration was observed. The measured PA signals and the fitted straight line are shown in (Figure 4). The smallest measured concentration was 20 ppbV for sulfur hexafluoride. As this function has a linear behavior we can extend this linearity to ppmv levels.

**Figure 5.**
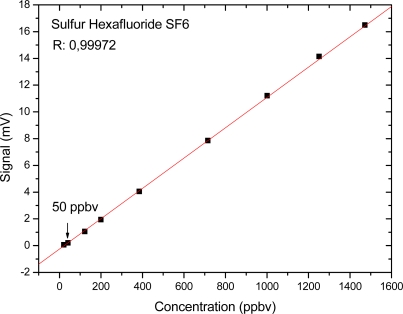
Calibration curve for sulfur hexafluoride.

The pulsed quantum cascade laser (QCL), delivering a power of 1.12 mW at the wavelength of 10.5 μm, was used to excite the sulphur hexafluoride molecules. To determine the detection limit of the system, a dilution of a standard mixture of 5 ppmv of SF_6_ in N_2_ was carried out. Small concentrations of the gas were synthesized by using two electronic mass-flow controllers, one for N_2_ (with full scale control of 200 sccm) and another for the investigated gas (SF_6_) (with full scale control of 50 sccm). The electronic mass-flow controllers were connected in parallel to the gas inlet of the photoacoustic cell, and the initial SF_6_ concentration of 5 ppmv was diluted with pure nitrogen (zero gas) down to the lowest concentrations detected by the system. The acoustic and the electronic noise were determined by blocking the laser light while keeping all other devices running. The value of the noise signal was typically 0.3 μV. As expected, a linear dependence of the photoacoustic signal on the sulfur hexafluoride concentration was found. The measured PA signals and the fitted straight line are shown in (Figure 5). The smallest measured concentration of sulfur hexafluoride was 50 ppbv. As this function has a linear behavior, we can extend this linearity to ppmv levels.

While the QCL provided a detection limit of 50 ppbv, the CO_2_ laser reached a limit of 20 ppbv, therefore proving to be more sensitive. However, pulsed quantum-cascade distributed-feedback (QC-DFB) lasers provide quasi room temperature operation, combined with a high spectral selectivity and sensitivity, real-time measurement capabilities, robustness, and compactness, whereas CO_2_ lasers are large, unstable and expensive in comparison. For this reason, QCLs are ideal for the development of compact trace gas analyzers that are also suitable for field measurements. We present an experimental setup where the QCLs is coupled to a differential photoacoustic cell that produces a photoacoustic signal with noise 100 times smaller (0.3 μV) when compared with the photoacoustic cell used in the experiment with CO_2_ laser (30 μV). With the development of QCLs with continuous emission, rather than pulsed, greater power is provided, and along with proper alignments to optimize the laser beam it will be possible to achieve detection limits comparable to those obtained with the CO_2_ laser.

For practical applications (industrial environments) that involve measurements in the atmosphere, the method proposed in this work must consider the presence of some chemical species that can alter the concentrations obtained for the SF_6_ gas, such as CO_2_ and water vapor. Thus one must use KOH and CaCl_2_ filters so as to eliminate the possible interference from those species [34]. The typical concentration of SF_6_ observed in the atmosphere is about 7 pptv, determined by NOAA (National Oceanic & Atmospheric Administration), on Mauna Loa, Hawaii, U.S.A. [35]. This value can be increased up to hundreds of ppbv to ppmv in certain processes and leaks in industrial environments.

## 4. Conclusions

The Photoacoustic Spectroscopy using CO_2_ and quantum cascade lasers has proved to be extremely sensitive and selective for the detection of sulfur hexafluoride. This methodology allows measurements in anthropogenic sources that can reach concentrations higher than 20 ppbv, when using the CO_2_ laser, and higher than 50 ppbv when using the pulsed quantum cascade distributed-feedback (QC-DFB) laser. These results are very promising and encourage further research.
